# British Medicinal Plants

**Published:** 1891-05

**Authors:** Alfred Heath

**Affiliations:** London, England


### BRITISH MEDICINAL PLANTS.

Alfred Heath, M. D., F. L. S., London, England.

### Order 21.—Oxalidaceæ.

*Oxalis acetocella* (Wood Sorrel).—A pretty little plant common in woods; it has white flowers, with purple veins; rarely the flowers are purple or blue. The plant is agreeably acid, and is good to quench thirst in fevers. A decoction of the plant has been given against scurvy and as a diuretic. It has been recommended in inflammatory, bilious, and putrid fevers. The leaves are sometimes applied externally in indolent scrofulous tumors. There is no proving.

### Order 22.—Linaceæ.

*Linum usitatissimum* (Common Flax).—An escape from cultivation. The seed of this plant is what is known as linseed. An excellent drink is made by boiling the seed in water. It is very useful in coughs and disorders of the chest. It is said to be excellent for gravel and stone. The crushed seed is commonly used for making "poultices," which are not unmixed blessings, and often do great mischief. Oil is also expressed from them, which is in general use for various purposes; the cake remaining is used for feeding cattle, etc. The fibre of this plant is made into linen.

*Linum catharticum* (Purging Flax).—A pretty little plant about six or eight inches high, found in dry, hilly pastures. This plant, as its name implies, is very purgative. It has been used as a cure for rheumatic pains and as a remedy for dropsy.

### Order 23.—Celastraceæ.

*Euonymus Europæus* (the Spindle Tree, Prickwood, Skewerwood).—The spindle tree is poisonous. The beautiful scarlet drupes which it bears in the autumn have been used as a dye. There does not appear to have been any use made of this tree in medicine until comparatively recent years. There is a proving of the drug in *Allen's Handbook.* It has been used in disturbances of the liver, biliousness, headache, constipation, in gastric derangements associated with albuminaria.

### ORDER 24.—RHAMNACEÆ.

*Rhamnus catharticus* (Buckthorn).—The berries of this tree have often been adulterated with alder berries, *Rhamnus frangula*, but are easily distinguished, the berry of the former having four seeds, and the latter two seeds only. Buckthorn berries have long been in considerable esteem as a cathartic, and celebrated in dropsies, rheumatisms, gout, etc. There is a short proving in Allen's *Materia Medica.*

*Rhamnus frangula* (Black Alder).—This and the last-mentioned are the only two trees of the order *Rhamnaceæ* found in this country. Decoctions made from the *fresh, green, inner bark* produce strong vomiting and griping pains in the stomach. The dried bark has been used as a remedy in dropsy and jaundice. It has been applied as a cure for the itch. The dried outer bark is said to be quite contrary in its action to the inner bark, binding the bowels and lessening immoderate fluxes. A decoction in vinegar is said to kill lice, cure scabs on the head, and dry up running humors. It is said to relieve some forms of toothache and to fasten teeth that are loose. The leaves are said to increase milk in cows, ease the pain in inflammations and swellings. When placed under the feet they are said to ease the pain and heat after walking. The freshly-gathered leaves strewed about a room are said to cleanse it of fleas. There is a short proving in Allen's *Materia Medica.*

### ORDER 25.—LEGUMINOSÆ.

*Ulex Europæus* (Furze, Whin, Gorse).—This plant is said to be useful in obstructions of the liver and spleen. A decoction of the flowers was at one time used in treating jaundice; it also acts as a diuretic, and is reputed as a remedy for gravel and stone. There is no proving.

*Genista tinctoria* (Dyers' Broom).—This plant has not been much used in medicine. It is laxative and diuretic, and has been used in the treatment of dropsy. It is esteemed in Russia as a cure for hydrophobia. It is used also as a dye.

